# F34. AUDITORY SENSORY GATING IN YOUNG ADOLESCENTS WITH EARLY-ONSET PSYCHOSIS: A COMPARISON WITH ADHD

**DOI:** 10.1093/schbul/sby017.565

**Published:** 2018-04-01

**Authors:** Cecilie Lemvigh, Jens Richardt Møllegaard Jepsen, Birgitte Fagerlund, Anne Katrine Pagsberg, Birte Glenthoj, Jacob Rydkjaer, Bob Oranje

**Affiliations:** 1 Center for Neuropsychiatric Schizophrenia Research, Center for Clinical Intervention and Neuropsychiatric Schizophrenia Research, University of Copenhagen; 2 Child and Adolescent Mental Health Center; 3 University Medical Center Utrecht, Holland/Center for Neuropsychiatric Schizophrenia Research, and Center for Clinical Intervention and Neuropsychiatric Schizophrenia Research, University of Copenhagen

## Abstract

**Background:**

Numerous studies have demonstrated impaired sensory gating in schizophrenia and this phenomenon has been proposed as a candidate biomarker for the disorder. Sensory gating is typically assessed during an auditory paired-click test commonly referred to as a P50 suppression paradigm. When two identical stimuli are presented, healthy subjects show a decrease in their neural response to the second stimulus, reflected in a decreased P50 amplitude, whereas schizophrenia patients on average show a much smaller decrease. So far, sensory gating has primarily been investigated in adult patients with schizophrenia, but gating disturbances have also been demonstrated in other illnesses, e.g. in schizotypal personality disorder, albeit less marked. Although the typical age of onset for schizophrenia is late adolescence to early adulthood, a sizable group of patients presents with psychotic symptoms during childhood or early adolescence. Manifestation of psychotic symptoms before the age of 18 is commonly referred to as early-onset psychosis (EOP). Various studies have reported a more severe course of illness and a poorer outcome in EOP compared to the adult-onset form of the disorder. In parallel, we expect more pronounced sensory gating deficits in EOP.

Impaired sensory gating may not be specific to psychosis, but rather a shared disturbance of neuropsychiatric disorders. Although symptoms of attention deficit hyperactivity disorder (ADHD) differ in many ways from those found in schizophrenia, there are common characteristics. Compared to schizophrenia, relatively few studies have investigated sensory gating in ADHD, and some report P50 gating deficits similar to those frequently found in patients with schizophrenia.

**Methods:**

We investigated P50 suppression in a large cohort of adolescents (12–17 years old) consisting of patients with either EOP (N=56) or ADHD (N=28) as well as age and gender matched healthy controls (N=72). In our paradigm two identical sounds (clicks) were presented separated by a 500ms interval. The amount of suppression was expressed as the ratio between the P50 amplitude of a subject’s response to the first click and his/her amplitude in response to the second click.

**Results:**

The EOP patients scored significantly higher on PANSS (positive, negative, general, and total PANSS scores) compared to both ADHD patients and healthy controls. However, there were neither significant group differences in raw P50 amplitude, nor in the gating ratios between young adolescents with EOP, ADHD and healthy controls.

**Discussion:**

This is the first study to investigate sensory gating in young adolescents with EOP. We found no P50 suppression deficits in these patients which, given the relatively large sample size in our study, cannot merely be ascribed to power issues. The results are in contrast with the majority of studies investigating sensory gating in schizophrenia and ADHD. However, the results are in agreement with earlier studies from our lab showing evidence of inconsistent P50 suppression deficits in two separate cohorts of adult, antipsychotic naïve, first-episode patients with schizophrenia. Based on our findings, P50 sensory gating cannot differentiate between young adolescents with EOP or ADHD, and deficient P50 suppression does not seem to be a valid biomarker for EOP.

